# Machine Learning Techniques for the Analysis of the Influence of Blood Gasometry Parameters on Acid–Base Homeostasis in Pediatric Patients

**DOI:** 10.3390/diagnostics15243166

**Published:** 2025-12-11

**Authors:** Maria Dybała, Alicja Bartkowska-Śniatkowska, Krzysztof Pietrzkiewicz, Anna Wiernik, Jowita Rosada-Kurasińska, Tomasz Piontek, Ariel Oleksiak, Andrzej Czyrski

**Affiliations:** 1 Poznan University of Medical Sciences, Department of Physical Pharmacy and Pharmacokinetics, Rokietnicka 3 Street, 60-806 Poznań, Poland; 2 Poznan University of Medical Sciences, Department of Pediatric Anesthesiology and Intensive Care, Szpitalna 27/33 Street, 60-572 Poznań, Poland; 3 Poznan Supercoputing and Networking Center, Jana Pawła II 10 Street, 61-139 Poznań, Poland

**Keywords:** acidosis, alkalosis, arterial blood gas, artificial neural networks, LASSO regression, pediatric intensive care unit

## Abstract

**Background/Objectives:** The study aimed to evaluate the most significant factors that impact arterial blood gas parameters: pH, pO_2_, pCO_2,_ and concentration of lactates. **Methods:** The study was a retrospective analysis of clinical data obtained from the patients’ records hospitalized at the Department of Pediatric Anesthesiology and Intensive Care. A total of 71 patients were enrolled in the study. A total of 479 measurements were performed for arterial blood, 41 were excluded. The analysis was performed for 438 results. The artificial neural network (ANN) regression models were applied, and the Least Absolute Shrinkage and Selection Operator (LASSO) regression was used. ANNs were built considering the following activation functions: hyperbolic tangent, linear, exponential, and logistic. The following three sets were separated: training, testing, and validation. In the case of LASSO regression, the regularization was applied, excluding insignificant variables from the model. Besides the machine learning techniques, the correlation between the variables was calculated. **Results:** The correlation coefficients for regression ANN models exceeded the value for testing set of 0.92. According to the sensitivity analysis, the most significant variable for pH was cCl^−^, for pO_2_ it was pO_2_/FiO_2_, for pCO_2_ it was Fshunt, and for concentration of lactates it was pH. In the case of LASSO regression for pH, the most significant factor was pCO_2_, for pO_2_ it was pO_2_/FiO_2_, for pCO_2_ it was cCl^−^, and for concentration of lactates it was pCO_2_. **Conclusions:** The results show the usefulness of machine learning methods in analyzing complex physiological relationships. Such techniques can help improve diagnostic accuracy and optimize therapeutic management in pediatric patients.

## 1. Introduction

Maintaining acid–base balance is one of the most crucial aspects of preserving the body’s homeostasis. This phenomenon has been extensively studied for over 70 years since the research of John Severinghaus and Leland Clark [1]. Nowadays, their groundbreaking achievements are part of the foundation of pathophysiology, which is vital for the understanding of patients’ conditions, not only in intensive care, but also in almost every other field of health studies. The body tightly controls the regulation of proper pH levels by adjusting the ventilation rate, buffers such as ${HCO}_{3}^{-}$, and renal mechanisms. pH should be kept within the range 7.35–7.45. Disturbances in acid–base balance can lead to serious clinical consequences. On the other hand, they are common in the intensive care units and may be divided into the following categories: metabolic (acidosis and alkalosis) and respiratory (acidosis and alkalosis). They contribute significantly to higher morbidity and mortality [2,3,4].

The mathematical formula, such as the Henderson–Hasselbalch equation, describes the relationship between pH and the concentration of an ionized salt and a nonionized acid molecule. The other is the Stewart approach, which states that whole body acid–base balance can be estimated quantitatively regarding pCO_2_, net strong ion charge, and total weak acids (albumins, inorganic phosphorus). It is regulated by the lungs, kidneys, gut, and liver [5].

There has been growing interest in using various machine learning techniques as analytical methods in recent years. These approaches enable rapid analysis of large clinical databases and modeling complex, nonlinear relationships between different physiological parameters that may be potentially interconnected. Such techniques include artificial neural networks (ANNs) and Least Absolute Shrinkage and Selection Operator (LASSO) regression. Neural network deep-learning models might be useful in identifying diabetic retinopathy, cardiovascular risk, or moles from melanomas [6].

The operation of ANN is modeled on the functioning of biological nerve cells. By enabling various activation functions and adjusting the network architecture, they allow for modeling complex relationships between individual parameters [7].

The study aims to evaluate the factors that influence the following parameters: pH, pCO_2_, pO_2,_ and lactate concentration (cLac) in arterial blood in patients from the pediatric intensive care unit. We integrated the following computational paradigms—specifically, ANN for their nonlinear modeling capabilities, LASSO regression for its robust feature selection and regularization properties. We also applied classical correlation for its foundational interpretability of linear relationships—to analyze the influence of various blood gasometry parameters on acid–base balance. To our knowledge, this is the first investigation to employ such a hybrid, multi-model analytical framework specifically designed to elucidate the relative contributions of blood gas variables in maintaining acid–base homeostasis. This innovative approach promises a nuanced and holistic understanding of pathophysiological mechanisms, which can ultimately guide more precise and personalized therapeutic interventions for patients with acid–base balance disorders.

## 2. Material and Methods

### 2.1. Characteristics of Patients and Analyzed Parameters

The conducted study was a retrospective one. It was based on the anonymized patients’ data extracted from patients’ records [8]. According to the statement of the Local Ethics Committee at Poznan University of Medical Sciences, approval was not necessary (S59/25).

The study cohort consisted of patients admitted to the Pediatric Intensive Care Unit at the Karol Jonscher Clinical Hospital of the Poznan University of Medical Sciences between 1 October 2024 and 31 March 2025. A total of 71 patients aged 0–17 years old were enrolled in the study. The mean age was 4.83 ± 5.27 years old (median 2 years old). The M/F ratio was 41/30. In total, 479 measurements were performed. A total of 41 measurements were excluded from the analysis—the Fshunt fraction was below 0, which indicates the measurement error. The analysis was performed for 438 arterial blood measurements. In the case of patients, there were no inclusion and exclusion criteria.

The following parameters were taken into consideration: FiO_2_—fraction of inspired oxygen, ctHb—total concentration of hemoglobin, sO_2_—saturation, cK^+^—concentration of potassium, cNa^+^—sodium concentration, cCl^−^—chloride concentration, cLac, Fshunt—fraction of the measured transpulmonary shunt, pO_2_, pCO_2_, pH, and pO_2_/FiO_2_. The dependent variables in the models were pO_2_, pCO_2_, pH, and cLac.

### 2.2. The Statistical Analysis

The correlation factor was estimated between the dependent variables, which were pO_2_, pCO_2_, pH, cLac, and the following variables: FiO_2_, ctHb, sO_2_, cK^+^, cNa^+^, cCl^−^, cLac, Fshunt, pO_2_, pCO_2_, pH, and pO_2_/FiO_2_. The statistical analysis was performed with Statistica 13.3 Software (Tibco Software Inc., Palo Alto, CA, USA).

### 2.3. Artificial Neural Network Modeling and Biological Testing

ANN is a computational approach capable of modeling nonlinear and intricate relationships among multiple physiological variables. It is advantageous in arterial blood gas analysis, where interactions between factors such as pH, pO_2_, pCO_2_, and cLac exhibit complex dependencies. Traditional linear models might fail to capture effectively. The flexibility of ANN to learn from data without assuming any specific parametric form allows the uncovering of hidden patterns and improves predictive accuracy in clinical settings [9].

The arterial blood gasometry results from patients described in Section 2.1 were analyzed with ANN. The multilayer perceptron (MLP) was applied. It is vastly applied in numerous practical regression and classification scenarios [10]. The applied ANN consists of the input, the output layer, and one hidden layer. The ANN was trained in feed-forward, backpropagation, and a supervised learning algorithm. The training was conducted in the following steps described by Imai et al. [11]: the provision of the data to the input layer, the obtained result in the output layer and evaluation of the error, backpropagation to the hidden layer, and the process starts again until the error takes the lowest value and 300 training epochs as the maximal number of trainings. The following activation functions for the hidden and output layers were considered during the ANN modeling: linear, exponential, logistic, hyperbolic tangent, and sinus. The dataset was divided into three subsets to prevent overfitting: training, testing, and validation. The training set contained 70% of the cases. Both testing and validation sets contained 15% of the cases each. The model quality was evaluated with the correlation coefficient value, which should approach the value of 1.0000 for training, testing, and validation set. The neural modeling was performed with Statistica 13.3 software (Tibco Software Inc., Palo Alto, CA, USA).

### 2.4. LASSO Regression Analysis

LASSO regression was used to account for the need to select variables and regularize the model. By imposing a penalty on the absolute magnitude of regression coefficients, LASSO effectively reduces insignificant predictors to zero, thereby eliminating redundant or irrelevant variables. This feature reduces the risk of overfitting, improves model interpretability, and facilitates the identification of factors that have the greatest impact on blood gas parameters.

The LASSO regression coefficients were estimated by minimizing the residual sum of squares subject to an L1 penalty on the coefficients, effectively shrinking some coefficients to zero and thus selecting a subset of predictors most relevant to each dependent variable. Model tuning was conducted using cross-validation to identify the optimal regularization parameter (λ), balancing model complexity and predictive accuracy. This approach allowed us to identify key predictors influencing each blood gas parameter while controlling for potential confounding effects of correlated variables.

The LASSO regression was applied to find the best predictors for the analyzed dependent variables pO_2_, pCO_2_, pH, cLac. The independent variables were FiO_2_, ctHb, sO_2_, cK^+^, cNa^+^, cCl^−^, cLac, Fshunt, pO_2_, pCO_2_, pH, and pO_2_/FiO_2_. It was calculated using Statistica 13.3 software (Tibco Software Inc., Palo Alto, CA, USA).

### 2.5. Key Steps in the Machine Learning Analysis

To create a risk factor classifier, we analyzed arterial blood gas data from 71 pediatric intensive care unit patients using two complementary modeling methods. First, we used ANN to evaluate the factors that influence key physiological variables (pO_2_, pCO_2_, pH, cLac). The ANN consisted of a single hidden layer, was trained using supervised backpropagation for up to 300 epochs, and was optimized by testing multiple activation functions. The dataset was divided into training, test, and validation subsets to minimize overfitting, and model performance was evaluated using correlation coefficients across all subsets.

Second, we used LASSO regression for variable selection and regularization. By applying the L1 penalty, LASSO reduced insignificant coefficients to zero and identified the most influential predictors among parameters such as FiO_2_, ctHb, sO_2_, cK^+^, cNa^+^, cCl^−^, cLac, Fshunt, pO_2_, pCO_2_, pH, and pO_2_/FiO_2_. Cross-validation was used to determine the optimal value of λ. The combination of these methods allowed us to construct a classifier that highlights the strongest risk factors while ensuring both prediction accuracy and interpretability.

The methodology consists of the following key steps:

Data Collection: retrospective extraction of clinical data from pediatric patients’ arterial blood gas records.

Data Preprocessing: cleaning the dataset.

Dataset Splitting: division into training (70%), testing (15%), and validation (15%) subsets to ensure robust model evaluation and prevent overfitting.

ANN Modeling: Construction of MLP structure with input layer (physiological variables), one hidden layer, and output layer (blood gas parameters). Training using feed-forward backpropagation with supervised learning. Testing different activation functions (linear, exponential, logistic, hyperbolic tangent, sinus). Training stops upon minimum error or maximum of 300 epochs.

LASSO Regression Analysis: application for variable selection and regularization to identify the most significant predictors.

Model Evaluation: performance measured by correlation coefficient, and identification of influential variables by sensitivity analysis.

Result Interpretation and Application: understanding physiological relevance, and potential clinical application in diagnostics and management.

## 3. Results

### 3.1. Gasometric Parameters

The results for the gasometric parameters analysis for arterial blood are presented in Table 1.

### 3.2. The Correlation Analysis

The correlation coefficients for the analyzed variables were calculated. The results for arterial blood are presented in Table 2.

### 3.3. Neural Network Modeling and Biological Testing

The ANNs are based on the MLP algorithm. The impact of the independent variables on the analyzed response was evaluated using sensitivity analysis. The value higher than 1.0000 implied the significance of the analyzed variable. A value lower than 1.0000 implies a lack of significance, and the variable should be removed from the model (Table 3).

#### 3.3.1. pH

In case of pH, the impact of the following variables was considered: FiO_2_, ctHb, pO_2_, pCO_2_, sO_2_, cK^+^. cNa^+^, cCl^−^, cLac, pO_2_/FiO_2_, and Fshunt. The best parameters for pH were obtained for the following network MLP 11-30-1. The correlation coefficient was 0.9322. It implies a strong correlation between the observed and simulated values. The network was found in the 50th epoch. The following activation functions were applied: hyperbolic tangent for the hidden layer and the exponential for the output layer.

**Figure 1:**
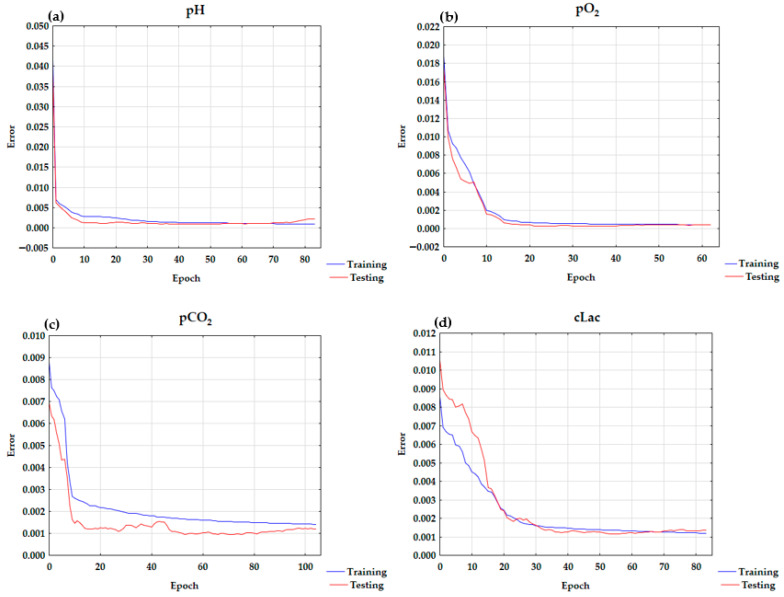
The learning curve charts for (**a**) pH, (**b**) pO_2_, (**c**) pCO_2_, and (**d**) cLac.

cCl^−^ and pCO_2_ exert the greatest influence on pH value. Subsequently, the following factors are ranked in order of their descending impact: cNa^+^, cLac, pO_2_/FiO_2_, sO_2_, Fshunt, FiO_2_, pO_2_, cK^+^, and ctHb. The results of the sensitivity analysis are presented in Table 3. The learning curve chart for pH is presented in Figure 1a.

#### 3.3.2. pO_2_

In pO_2_, the impact of the following variables was considered: FiO_2_, ctHb, pH, pCO_2_, sO_2_, cK^+^, cNa^+^, cCl, cLac, pO_2_/FiO_2_, and Fshunt. The best parameters for the neural network were observed for MLP 11-13-1 with the logistic function in both the hidden and output layer. The correlation coefficient was 0.9884. It implies high correlation between the observed and simulated data for the analyzed network. The network was found in the 32nd epoch.

The greatest influence on pO_2_ was exerted by pO_2_/FiO_2_. The other factors are ranked in descending order of their impact: FiO_2_, Fshunt, sO_2_, pH, pCO_2_, cCl^−^, cNa^+^, cK^+^, cLac, and ctHb. The learning curve for pO_2_ is presented in Figure 1b. The results of the sensitivity analysis are presented in Table 3.

#### 3.3.3. pCO_2_

For pCO_2_, the following independent variables were taken into consideration: FiO_2_, pH, ctHb, pO_2_, sO_2_, K^+^, Na^+^, Cl^−^, cLac, pO_2_/FiO_2_, and Fshunt. The best network parameters were observed for MLP 11-28-1 with the logistic function as an activation function in both the hidden and output layers. The correlation coefficient was 0.9261. It implies high correlation between the observed and simulated data for the analyzed network. The network was found in the 53rd epoch.

The greatest influence on pCO_2_ was observed for Fshunt. The other factors were in the following descending order of importance: pO_2_/FiO_2_, cCl^−^, pH, cNa^+^, FiO_2_, cLac, sO_2_, pO_2_, ctHb, and cK^+^.

The learning curve chart for pCO_2_ is presented in Figure 1c. The results of the sensitivity analysis are presented in Table 3.

#### 3.3.4. cLac

For neural network modeling of the dependent variable cLac for data obtained from arterial blood analysis: FiO_2_, pH, ctHb pO_2_, pCO_2_, sO_2_, K^+^, Na^+^, cCl^−^, pO_2_/FiO_2,_ and Fshunt were used as quantitative independent variables.

The best parameters for pH were obtained for the following network MLP 11-7-1. The correlation coefficient was 0.9327. The value confirms the high correlation between the observed and simulated data. The network was found in the 53rd epoch. The activation function was the hyperbolic tangent for the hidden and output layers.

The ANN analysis indicated that pH was the most significant factor influencing cLac. Subsequently, the following factors are ranked in order of their descending impact: pCO_2_, Fshunt, pO_2_/FiO_2_, FiO_2,_ cK^+^, cCl^−^, sO_2_, pO_2_, cNa^+^, and ctHb. The learning chart curve for cLac is presented in Figure 1d. The results of the sensitivity analysis are presented in Table 3.

### 3.4. LASSO Regression

**Figure 2:**
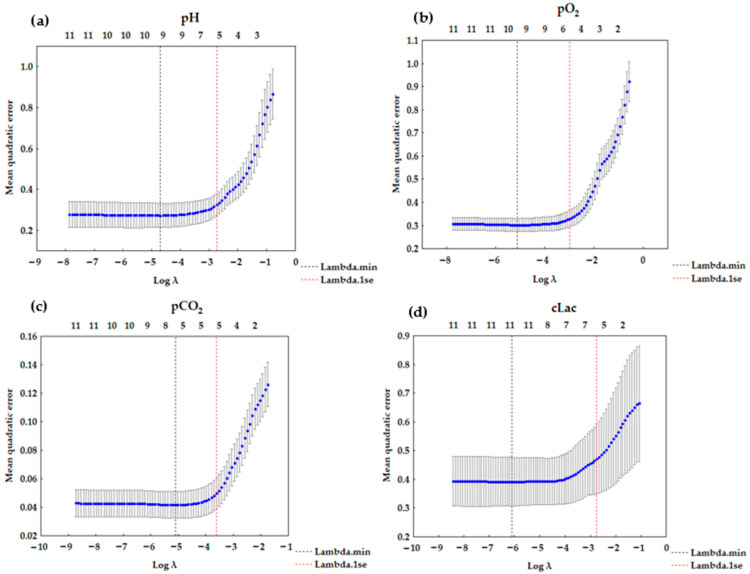
LASSO regression graph (**a**) for pH—6 variables (λ = 0.0645); (**b**) for pO_2_—6 variables (λ = 0.0504); (**c**) for pCO_2_—5 variables (λ = 0.0270); (**d**) for cLac—6 variables (λ = 0.0642).

**Figure 3:**
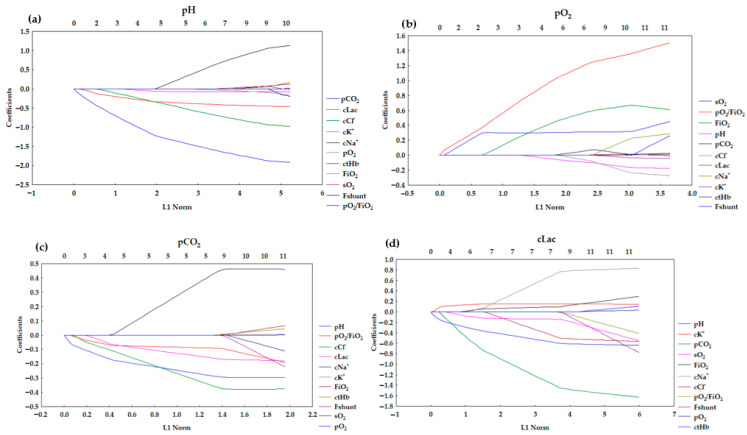
Lasso coefficient profiles of 11 variables for (**a**) pH, (**b**) pO_2_, (**c**) pCO_2_, (**d**) cLac.

The results for LASSO regression are presented in Table 4. LASSO (or any regression) shows association, not causation. The number of corresponding variables for pH, pO_2_, pCO_2_, and cLac screened out by the model is listed in Figure 2a–d. A LASSO regression model was used to build a risk factor classifier (Figure 3a–d). The low values of L1 indicate strong regularization (high λ values). High values of L1 indicate weak regularization, and low λ values. The analysis indicated the significant factors for the analyzed dependent variables. In the case of pH, (λ = 0.0645) and the following variables were significant: pCO_2_, pO_2_, cK^+^, cNa^+^, cCl^−^, and cLac. For pO_2_ (λ = 0.0504), FiO_2_, pH, pCO_2_, sO_2_, cCl^−^, and pO_2_/FiO_2_ were included in the model. For pCO_2_ (λ = 0.0270), the following variables were significant: pH, cNa^+^, cCl^−^, cLac, and pO_2_/FiO_2_. In the case of cLac (λ = 0.0642), the following parameters were included in the model: FiO_2_, pH, pCO_2_, sO_2_, cK^+^, and cNa^+^.

## 4. Discussion

The study aimed to apply machine learning techniques such as ANN and LASSO regression in analyzing factors affecting arterial blood gas parameters in pediatric patients in the intensive care unit. The analysis was performed for the following dependent variables: pH, pO_2_, pCO_2_, and cLac.

### 4.1. pH

In the ANN analysis, model with high testing quality, minimal differences between testing and learning quality, and low error was obtained. The structure of the ANN was as follows: MLP 11-30-1 model with a hyperbolic tangent in the hidden layer and an exponential function in the output layer. As seen in the learning chart in Figure 1a, the overtraining was not observed. The correlation coefficient was 0.9322. It confirms the model is a good fit.

A sensitivity analysis was conducted to assess the influence of each independent variable on the response. This analysis presents the influence of each parameter hierarchically (Table 3). According to the sensitivity analysis (Table 3), cCl^−^ strongly influences pH (4.0085) as well as pCO_2_ (3.9872). This implies that Cl^−^ is a key anion strongly affecting plasma pH [12]. This is related to the Stewart model but cannot be easily explained using the Henderson–Hasselbach equation. Hyperchloremia is a common contributor to metabolic acidosis in critically ill patients. In pediatric cases of diabetic ketoacidosis, hyperchloremia emerges as the predominant metabolic factor driving acidosis after 12 h of treatment. It accounts for approximately 98% of the base deficit and frequently delays the resolution of metabolic acidosis in these patients. Hyperchloremic acidosis may lead to hypotension or renal dysfunction [13,14].

The second influential factor is pCO_2_. It affects pH through the bicarbonate buffer system and follows the Henderson–Hasselbalch equation. It is a hydrogen producer in H_2_CO_3_ reaction [12]. cNa^+^ is ranked in third place (2.1979). Sodium is a major plasma cation affecting the ion difference and pH [12]. In the case of cLac, the effect is moderate (1.9464). It reflects its role as an acid–base status indicator, which increases in acidosis [15]. This interpretation aligns with recent research demonstrating that the independent variables of pH include electrolytes (Na^+^, K^+^, Cl^−^), CO_2_, lactate, and other factors, all evaluated separately for accurate acid–base assessment [12]. Other variables such as pO_2_, cK^+^, sO_2_, pO_2_/FiO_2_ ratio, FiO_2_, Fshunt, and ctHb have smaller but measurable impacts (all <1.5), indicating secondary or indirect influences on pH likely through metabolic or respiratory interactions.

The correlation analyses for the arterial blood parameters, presented in Table 2, showed that pH has the strongest negative correlation with pCO_2_. This means that an increase in pCO_2_ leads to a decrease in pH (respiratory acidosis), while a decrease in pCO_2_ results in an increase in pH (respiratory alkalosis). It may be explained by the equation:

$$CO_{2}+H_{2}O\to H_{2}CO_{3}\to H^{+}+HCO_{3}^{-}$$

The increase in pCO_2_ shifts the reaction to the right, which results in an increase in H^+^ ions. This relationship is explained by the Henderson–Hasselbalch equation, which describes how pH depends on the concentration of hydrogen ions and carbon dioxide in blood plasma. The increase in pCO_2_ causes the buffer equation to shift towards producing H^+^ ions, lowering the blood pH and making it more acidic. On the other hand, the decrease in pCO_2_ raises pH, making the blood more alkaline [16,17,18].

The negative correlation was observed between pH and Fshunt (Table 2). Which could be related to the amount of carbon dioxide that bypasses the lungs and therefore remains in the blood, causing an exacerbation of acidosis. However, pH often decreases in the state of significant shunting (as Fshunt increases). It results in acidemia. This results from the impaired gas exchange, which is an immediately life-threatening condition, and the accumulation of CO_2_, which leads to lower pH. However, the compensatory mechanisms such as renal compensation or increased compensatory drive can influence it in the patient [19,20]. It is worth noting that these mechanisms are significantly slower and metabolic compensation may take hours or days or even longer.

Correlation analysis of the effect of FiO_2_ on pH revealed a negative correlation between these parameters (Table 2). The correlation reflects the severity of respiratory failure, not the pH regulation by FiO_2_ itself.

A positive correlation was shown between sO_2_ and pH (Table 2). This is directly related to the Bohr effect, whereby a decrease in pH reduces oxygen binding to hemoglobin (Hb), thereby lowering sO_2_ [21].

Our study shows a negative correlation between pH and cK^+^ and cCl^−^ (Table 2). The low pH (acidosis) results in a higher cK^+^. A similar trend is observed for cCl^−^, which causes hyperchloremic acidosis. In our studied population, the observed correlation between pH and cNa^+^ was negative. Iatrogenic hypernatremia may be caused by an extensive use of normal saline. In these situations, it is accompanied by a more pronounced increase in chloride concentration, and the net effect would be metabolic acidosis. Hypernatremia can accompany both alkalosis and metabolic acidosis. The more frequent condition is alkalosis. However, hypernatremia may be observed alongside metabolic acidosis in specific clinical situations, such as diabetic ketoacidosis, which may be combined with a hyperosmolar hyperglycemic state. The effect on pH is indirect and depends on other compensatory mechanisms (such as water loss or ion shifts in broader water and electrolyte metabolism) [22,23,24].

Lactates are formed in the body mainly by anaerobic glycolysis. According to the Stewart model, they constitute a significant part of the measurable strong anions. The production of lactates increases H^+^ concentration. It leads to decreased pH, which results in a negative correlation (−0.421) (Table 2).

The LASSO regression eliminated the non-significant values to the model. In our analysis it indicated pCO_2_, pO_2_, cK^+^, cNa^+^, cCl^−^, and cLac (Table 4). The largest negative values for the coefficients were noted for pCO_2_ and for cCl^−^. The signs of the coefficients in the LASSO regression were consistent with those of the correlation coefficients, except for cNa^+^. The correlation analysis indicated a negative correlation (Table 2), and the LASSO regression indicated a positive coefficient value for cNa^+^ (Table 4).

In the study of Gucyetmez et al., the multivariate analysis indicated that pH was significantly increased by Na^+^ and K^+^ [12]. The coefficient value for LASSO regression for cNa^+^ was 0.3990, indicating a strong impact. In the case of cK^+^, the coefficient was negative and took a low value (Table 4). LASSO regression is a model that considers multiple variables simultaneously. The LASSO regression coefficient results from optimization with an L1 penalty, which can reduce some coefficients to zero or change their sign relative to simple correlation. This is especially true when the variables are closely correlated or interdependent. Thus, in a multivariate regression model, regression may attribute a positive effect to a variable on the dependent variable despite a negative direct correlation with it. This can be indirectly explained by correlation, referring to two variables. Considering regularization and interdependence, LASSO regression looks for the best fit, which can change the sign of the regression coefficient. Positive effect of sodium on pH—an increase in sodium is associated with an increase in pH (a change in acidity/alkalosis). This may reflect, for example, compensatory mechanisms of water–electrolyte balance and the influence of sodium on ionic balance (the alkaline effect of sodium) [25].

The negative coefficient for pCO_2_ (−1.4616) indicates that an increase in pCO_2_ results in a decrease in pH, which results in acidosis (Table 4). In the case of cCl^−^, an increase in chloride levels causes a compensatory decrease in pH (e.g., hyperchloremic acidosis) [26]. The negative coefficient for cLac proves that the decrease in pH results from increased cLac (Table 4). Which—again—can be easily explained with Stewart’s approach, but not the Henderson–Hasselbach equation. The negative coefficient indicates that an increase in blood cK^+^ is associated with a slight decrease in pH, i.e., acidosis. Acidosis can lead to the shift in potassium from cells to the extracellular space (hyperkalemia) [27].

The pH model (Figure 3a) presented distinct negative associations for pCO_2_, cLac, and cCl^−^ with their coefficients decreasing rapidly. The coefficients for these variables start to change right after the L1 starts to grow, which implies that they are the strongest predictors. Other predictors showed relatively stable or weak contributions. It suggests that pH regulation is dominantly affected by respiratory (pCO_2_) and metabolic (cLac) acid–base disturbances. The overall pathway is characterized by early shrinkage of key variables with minor contributions from auxiliary predictors.

### 4.2. pO_2_

The MLP 11-13-1 model has a logistic activation function for both the hidden and output layers. The learning curve is presented in Figure 1b. The correlation coefficient was 0.9884.

**Figure 4:**
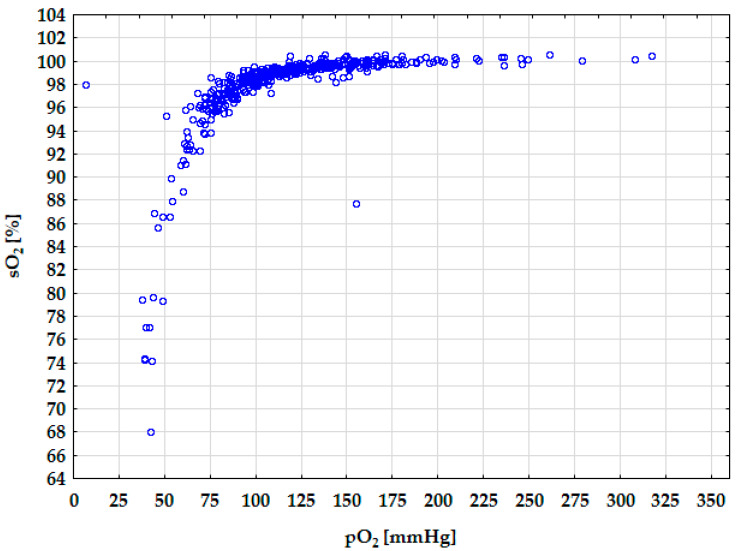
Diagram of the hemoglobin dissociation curve.

For the pO_2_ variable, the impact of the pO_2_/FiO_2_ ratio was the most influential (29.7513) (Table 3). The pO_2_/FiO_2_ ratio directly reflects oxygenation efficiency and lung function, which explains the highest impact. Villar et al. [28] indicated that pO_2_/FiO_2_ beside positive end-expiratory pressure (PEEP) at 24 h after moderate/severe acute respiratory distress syndrome (ARDS) appear to be critical for distinguishing distinct ARDS subgroups. The other factor is FiO_2_ (22.6468). It is the oxygen concentration that the patient inhales, which directly affects arterial oxygen pressure. Fshunt is also a significant factor (2.8196). It reflects the amount of blood bypassing oxygenation in the lungs, thus decreasing pO_2_. Other variables have sensitivity values close to 1, indicating minor or baseline influence on pO_2_ in this model context. The relationship between the sO_2_ and pO_2_ is presented in Figure 4. The graph’s trend line corresponds to the hemoglobin dissociation curve found in the literature [16]. It demonstrates the good fit of the obtained networks. When pO_2_ reaches values above 75–83 mmHg, the sO_2_ parameter is greater than 95%. Conversely, when pO_2_ is below 75 mmHg, sO_2_ decreases, indicating a blood oxygen-carrying capacity decline.

The correlation analysis of pO_2_ variable in Table 2 showed that the parameters showing the highest positive correlation for arterial blood pO_2_ are pO_2_/FiO_2_ (0.591) and sO_2_ (0.567) (Table 2). The highest negative correlation was observed for Fshunt (−0.533). The positive correlation between pO_2_ and sO_2_ implies that with an increase in pO_2_, the amount of oxygen dissolved also increases, increasing sO_2_. In the case of pO_2_/FiO_2_, if FiO_2_ is set as a constant value, the increase in pO_2_ results in the increase in pO_2_/FiO_2_. For FiO_2,_ a negative correlation was observed with pO_2_ [29,30]. Mayor et al. proved that Fshunt negatively correlates with admission oxygen saturation [31]. Our study confirmed that the observed correlation between pO_2_ and Fshunt is negative. As the Fshunt fraction increases, more deoxygenated blood bypasses ventilated alveoli, thereby reducing arterial pO_2_ due to a higher proportion of deoxygenated blood entering systemic circulation [31,32]. In the case of pO_2,_ the negative correlation is observed with pCO_2_, which is caused by the fact that the removal of pCO_2_ leads to higher pO_2_ and an increase in alveolar ventilation.

The LASSO regression indicated that the significant variables for the model were pH, sO_2_, FiO_2_, pO_2_/FiO_2_, pCO_2,_ and cCl^−^. For FiO_2,_ the positive value of the coefficient was observed for LASSO regression (Table 4), contrary to the negative correlation (Table 2). The positive coefficient for FiO_2_ implies that increased FiO_2_ results in higher pO_2_. In the case of the correlation analysis, the coefficient was negative (−0.074) (Table 2). However, this correlation was weak. LASSO regression is a multivariate model that simultaneously considers multiple variables and their interdependence and applies regularization. In the LASSO model, the FiO_2_ coefficient is interpreted assuming that other variables in the model remain constant. The presence of collinearity and complex relationships may cause the bivariate correlation to differ from the coefficient obtained in multivariate regression. In the case of correlation analysis, the simple relationship is analyzed without control of the other variables. The other independent variables in the model are considered in the case of LASSO regression. The bivariate correlation does not reflect complex relationships and indirect effects that are only visible in a multivariate regression model.

The highest positive coefficient value for pO_2_ for LASSO regression was noted for pO_2_/FiO_2_ (1.0699) (Table 4). It implies that this variable has the greatest impact on pO_2_. The positive coefficient is observed for FiO_2_ (0.4827) and sO_2_ (0.3066). It indicates that increased saturation results in increased pO_2_. The saturation of hemoglobin with oxygen accompanies the high pO_2_. They are included in the model. It means that blood with a higher pH (alkalosis) can cause an increased affinity of hemoglobin for oxygen (a leftward shift in the O_2_-Hb dissociation curve, which means that the Hb has higher affinity to oxygen), which may lead to a slight decrease in the arterial blood pO_2_ at the same saturation level. It is caused by the Bohr effect. Negative coefficients for several variables emerge but remain low—for pH (−0.0640) and for cCl^−^ (−0.0115).

In clinical practice, FiO_2_ often correlates with disease severity, which may contribute to decreased pO_2_ (a negative association observed in linear correlation). This relationship reflects that patients with more severe illness frequently require higher FiO_2_, while their arterial oxygen partial pressure (pO_2_) tends to decrease, usually due to impaired respiratory function (examples of an exception would be cyanotic cardiac defects or patients on ECMO). However, after considering saturation, pH, and pO_2_/FiO_2_ ratio, which are key mediators and covariates, the effect of FiO_2_ alone on pO_2_ is positive. The role of regularization in LASSO regression causes the coefficients to be “shrunk,” better reflecting the realistic influence of variables in a multivariate model. In clinical practice, FiO_2_ often correlates with disease severity, which may contribute to decreased pO_2_ (a negative association observed in linear correlation). This relationship reflects that patients with more severe illness frequently require higher FiO_2_, while their arterial pO_2_ tends to decrease due to impaired respiratory function.

The profile presented in Figure 3b is differentiated by a markedly positive association for pO_2_/FiO_2_. FiO_2_ and sO_2_ also rise, but not as significantly as for pO_2_/FiO_2_. However, the profiles for pO_2_/FiO_2_ and sO_2_ start to grow right after the L1 starts to change. This pattern is consistent with the physiology of oxygenation, primarily determined by oxygen delivery and saturation. Other variables contribute marginally.

### 4.3. pCO_2_

The MLP 11-28-1 model with a logistic activation function for the hidden and output layers best describes pCO_2_ in arterial blood. The correlation coefficient was 0.9261. The learning curve chart is presented in Figure 1c.

The sensitivity analysis for ANN indicated that Fshunt was the most significant factor (8.8687) influencing pCO_2_ in arterial blood (Table 3). Wagner et al. investigated whether the identification of pulmonary vascular obstruction is possible, when pneumonia is present at the same time [33]. In this study, the measurements of alveolar (exhaled) pO_2_ and pCO_2_, and arterial pO_2_ and pCO_2_ (from a blood sample) were collected. The values were converted to the physiological shunt and physiological dead space. The results in our study indicated and confirmed the impact of Fshunt on pCO_2_.

pO_2_/FiO_2_ is ranked in second place (4.5329) (Table 3). A low pO_2_/FiO_2_ indicates impaired gas exchange, which often correlates with changes in pCO_2_, since carbon dioxide elimination is closely linked to alveolar ventilation. cCl^−^ is in third place (3.9112). Cl^−^ levels are important for maintaining acid–base balance, which is closely linked to blood CO_2_ levels. Blood pH was ranked fourth (3.5341), which is strongly related to pCO_2_ because CO_2_ dissolved in plasma forms carbonic acid, affecting the blood’s acidity. The ANN analysis indicated cNa^+^ (2.2918) and FiO_2_ (2.1653) had a significant impact, as did cLac (1.6521). cLac is a critical parameter in emergencies. High cLac levels can indicate metabolic acidosis, which affects respiratory compensation and pCO_2_ levels. The remaining variables: sO_2_ (1.5504), pO_2_ (1.1285), ctHb (1.1140), cK^+^ (1.0577) have a lower but still significant influence on pCO_2_. cNa^+^ and cK^+^ concentrations relate to fluid balance and acid–base status. sO_2_, FiO_2_, pO_2_, and ctHb provide context on oxygen delivery and respiratory status, which may indirectly affect CO_2_ retention and elimination. The ANN sensitivity analysis shows that impaired lung function (high Fshunt, low pO_2_/FiO_2_) and acid–base balance parameters (cCl^−^, pH) are the primary drivers influencing pCO_2_ levels in the model. This aligns well with clinical physiology, where ventilation–perfusion (V/Q) mismatch and compensatory respiratory changes to acid–base disturbances govern arterial CO_2_. Secondary variables related to oxygenation and electrolytes modulate these effects to a lesser extent. All variables considered were significant for the model—the value of the factor exceeded 1.0000.

According to the correlation analysis, pCO_2_ is strongly influenced by pH. A negative correlation was observed (−0.489) (Table 2). The negative correlation was observed also for cCl^−^, pO_2_, sO_2_, pO_2_/FiO_2_, and cLac. A positive correlation was observed for Fshunt and FiO_2_. The decrease in cCl^−^ resulting from an increase in pCO_2_ is caused by the chloride shift, which is one of the mechanisms of pH regulation [34]. The positive correlation between Fshunt and pCO_2_ is caused by the fact that as Fshunt increases, pCO_2_ also increases. The increased Fshunt can lead to hypoxic blood [16]. In our study, a positive correlation was observed between pCO_2_ and FiO_2_. It might be explained that in patients with chronic CO_2_ retention, the respiratory drive may rely more on hypoxia than hypercapnia. Administering high FiO_2_ reduces hypoxic drive, leading to hypoventilation and retention of CO_2_. It raises pCO_2_ levels. In addition, high FiO_2_ can reduce areas of hypoxic pulmonary vasoconstriction, increasing blood flow to poorly ventilated alveoli. It worsens V/Q mismatch and leads to increased CO_2_ retention. The positive correlation might also be explained by oxygen administration displacing CO_2_ from hemoglobin (the Haldane effect), increasing the amount of dissolved CO_2_ in the blood, and thus measuring pCO_2_ [35,36,37]. The normal blood pH is in the range of 7.35–7.45. Increased pCO_2_ (hypercapnia) means decreased alveolar ventilation and leads to respiratory acidosis (decreased pH), while decreased pCO_2_ (hypocapnia) leads to respiratory alkalosis (increased pH).

The results for LASSO regression indicated that the pH, cNa^+^, cCl^−^, cLac, and pO_2_/FiO_2_ are significant to the model (Table 4). The negative values of the coefficients for the following variables imply that higher values for blood pH (more alkalotic), cCl^−^, cLac (reflecting metabolic acidosis), pO_2_/FiO_2_ (better oxygenation) are associated with lower pCO_2_. Higher cNa^+^ is associated with higher pCO_2_ (positive correlation). The coefficients for pCO_2_ exhibit pronounced negative associations for pH, cCl^−^, and cLac, echoing the interplay between respiratory and metabolic compensation (Figure 3c). The coefficient shrinkage is less abrupt. It suggests a more distributed dependence on multiple acid–base parameters. However, it is still physiologically consistent.

### 4.4. cLac

The MLP 11-7-1 model with the hyperbolic tangent for both hidden and output layers best describes cLac. The correlation coefficient was 0.9327.

The sensitivity analysis conducted for the ANN indicated that the most significant factors that influence cLac are pH, pCO_2_, Fshunt, pO_2_/FiO_2_, FiO_2_, and cK^+^ (Table 3). The remaining variables were significant to the model. However, their impact was not as significant as in the case of the abovementioned ones. The strongest influence on cLac is blood pH (value 3.0156). This reflects the close physiological link between cLac levels and acid–base status. Elevated cLac often accompanies metabolic acidosis, and changes in pH affect cellular metabolism and lactate production. Lactate accumulation contributes to lowering pH, while acidosis may also impair lactate clearance. pCO_2_ (2.4469) influences cLac, likely through its effects on acid–base balance and tissue perfusion. Elevated pCO_2_ (respiratory acidosis) may exacerbate metabolic acidosis, affecting lactate metabolism. Conversely, lactate accumulation can stimulate hyperventilation, reducing pCO_2_ as a compensatory mechanism. cK^+^ is also significant (1.5499). It is closely tied to cellular metabolism and acid–base balance. Acidosis and increased lactate often lead to potassium shifts from intracellular to extracellular fluid, raising serum potassium levels. Thus, cK^+^ changes and lactate concentrations are physiologically interrelated. Fshunt (2.0705), pO_2_/FiO_2_ (1.8647), and FiO_2_ (1.8644) have a similar impact. Inspired oxygen fraction and shunt fraction relate to tissue oxygen delivery. Hypoxia or inadequate oxygenation (high shunt, low FiO_2_) promotes anaerobic metabolism and elevated lactate production. These variables reflect the respiratory and circulatory status that influence lactate generation. In the case of sO_2_ and pO_2_, oxygen saturation and oxygen partial pressure impact oxidative metabolism. Lower oxygen availability results in anaerobic glycolysis, increasing lactate production [38].

Correlation analysis of the cLac variable revealed that pH primarily influences it (Table 2). The correlation between these parameters was discussed earlier in the context of pH. Negative correlations were also observed between cLac and sO_2_, pCO_2_, and pO_2_/FiO_2_. According to a study by Bisarya et al. on 2062 patients, the correlation between cLac and sO_2_ was strong only in patients at or below the critical oxygen delivery threshold, and only 3% of patients met this criterion. In the general population, cLac is a poor predictor for sO_2_ [39]. For larger populations, these parameters do not correlate with each other.

The positive correlation with cLac was observed for cK^+^, Fshunt, FiO_2_, cCl^−^, and cNa^+^. In the case of lactic acidosis, there is an elevated cLac in the blood, resulting in a decrease in pH (metabolic acidosis). It causes the movement of K^+^ ions from cells into the extracellular space, resulting in hyperkalemia [40]. Toledo et al. noted a positive correlation between cLac concentration and cCl^−^ [41]. Hyperchloremia and hyperlactemia contributed to metabolic acidosis. The study by Barker has shown that a high saline supply can cause hyperchloremia, leading to acidosis, which may favor higher cLac in states of perfusion disorders [26]. The positive correlation between cLac and Fshunt implies that increased Fshunt results in decreased oxygenation caused by the blood bypass of the alveolar oxygenation sites. That results in the increased anaerobic metabolism–lactate production. It causes the elevated cLac, which results from increased Fshunt [42]. The positive correlation between cNa^+^ and cLac may result from the administration of intravenous solutions containing both lactate and sodium. The increase in both patients’ parameters is more often related to severe metabolic disorders and treatment (sodium lactate-containing infusions) than to a direct, interdependent mechanism. cLac is most strongly influenced by parameters related to acid–base balance (pH, pCO_2_) and cellular electrolyte status (K^+^), reflecting metabolic disturbances typically present in anaerobic metabolism and tissue hypoxia. Additionally, variables related to oxygen delivery and respiratory function (FiO_2_, Fshunt, sO_2_, pO_2_) significantly affect lactate levels by modulating the balance between aerobic and anaerobic metabolism.

The LASSO regression indicated that the following variables were significant to the model: FiO_2,_ pH, pCO_2_, sO_2_, cK^+^, and cNa^+^ (Table 4). The results of the LASSO regression imply that the growth in FiO_2_ is associated with a slight increase in cLac. It may reflect that patients requiring more oxygen have more severe metabolic disturbances. In cK^+^, hyperkalemia is associated with higher cLac, which is linked to the acute metabolic states (hyperkalemia in acidosis). In the case of cNa^+^, the coefficient is lower, which implies that higher sodium may accompany dehydration or electrolyte imbalances that accompany higher cLac. The negative correlation is observed in pH, which is physiologically consistent—higher pH (alkalosis) is associated with lower cLac. A strong negative correlation is observed for pCO_2_. In the case of sO_2_, negative correlation was also observed. It implied that higher sO_2_ relates to lower cLac. It is consistent with less hypoxia and better tissue perfusion. The cLac model stands out for complex coefficient dynamics and broad divergence from zero. Multiple variables, including FiO_2_, pH, pCO_2_, sO_2_, cK^+^, and cNa^+^, emerge as significant predictors, with both positive and negative associations. Coefficient paths traverse a wider range compared to the other parameters, denoting a multifactorial character and less dominance by a single predictor (Figure 3). This reflects the integrative nature of lactate as a marker of systemic metabolic stress, respiratory dysfunction, and electrolyte imbalance. The LASSO regression plot for cLac (Figure 3d) is visibly different from the other three (pH, pO_2_, pCO_2_) (Figure 3a–c) because the pattern of coefficient selection and shrinkage is distinct: multiple variables show strong negative or positive coefficients that diverge more rapidly as the L1 norm increases. The coefficients reach substantial values compared to the other parameters analyzed. This suggests that cLac may have a more complex, multifactorial relationship with the predictors in the model, resulting in more prominent regularization effects and variable selections. This pattern may reflect underlying clinical realities: blood lactate levels are influenced by diverse metabolic, respiratory, and circulatory processes, often making it more difficult to model sparsely with LASSO, whereas pH, pO_2_, and pCO_2_ can sometimes be predicted with fewer key variables due to their dominant physiological mechanisms.

### 4.5. Clinical Impact

The application of ANN and LASSO regression in this study offers significant enhancement in the decision-making process. ANNs can model complex nonlinear relationships that are attributed to the clinical data. The properly trained models may accurately predict the outcome based on the provided data. This capacity allows for the earlier identification of physiological abnormalities and more precise monitoring of the patient’s state of health. They help to stratify the risk and mitigate possible complications. In the study by Gray et al., the prediction of patients at low risk of critical postoperative adverse events can be made based on an individual level due to the application of machine learning models [43]. It results in support for the clinical decision-making process.

LASSO regression, by employing regularization to penalize less relevant features, effectively reduces model complexity and multicollinearity. It is useful in selecting the most pertinent clinical variables. It facilitates the generation of simpler and more interpretable models that highlight the key determinants impacting patient parameters. Clinicians benefit from clearer insights into critical physiological factors. It may streamline diagnostic evaluation and optimize treatment strategies [44]. The integration of such techniques with patients’ treatment aligns with the emerging shift towards precision medicine, where timely, data-driven decisions can significantly improve the outcomes of therapy.

### 4.6. Challenges and Limitations

ANNs demonstrated their potential in modeling complex physiological relationships. They provide accurate predictions and practical insights into patient monitoring and therapeutic management in pediatric intensive care [43,45]. LASSO regression complements ANN by enabling robust feature selection, improving model interpretability, and directing clinical attention to key variables that influence arterial blood gas parameters.

However, translating such models into routine clinical use is challenging. A main limitation is that the model must be thoroughly clinically validated in diverse patient populations. It is essential for ensuring generalizability, reducing bias, and confirming clinical utility before widespread adoption [46]. The integration of such models requires real-time data acquisition and processing capabilities. It demands robust electronic health record systems and interoperability infrastructures. That might be a potential obstacle because many healthcare environments lack such an infrastructure [47].

The other very important issues are ethical and operational challenges. They include maintaining patient privacy, ensuring the explainability of the generated ANN models, and ensuring trust and acceptance by physicians by providing understandable risk assessments and recommendations [48]. Furthermore, continuous monitoring and updating the model is crucial to adapt it to changing clinical practices and patient populations, while preventing model degradation over time.

Overall, although ANN and LASSO provide an effective framework for improving clinical decision support, their successful implementation requires interdisciplinary collaboration between researchers, data scientists, IT specialists, and regulators to overcome these barriers and fully realize their potential to improve patient care.

### 4.7. Biological Testing

Biological testing was performed using ANN to model the following acid–base balance parameters—pH, pO_2_, pCO_2_, and cLac—based on arterial blood data. For each parameter, multiple relevant physiological and biochemical variables were included as inputs to MLP models. The network architectures, activation functions, and training epochs were optimized to achieve high predictive accuracy, evidenced by correlation coefficients typically exceeding 0.92 in for testing phase.

The ANN models demonstrated strong correlations between observed and predicted values, confirming their robustness. Variable importance analysis revealed physiologically plausible influences: cCl^−^ and pCO_2_ most strongly affected pH; pO_2_/FiO_2_ ratio was the main determinant of pO_2_; Fshunt fraction had the greatest impact on pCO_2_; and pH was the dominant factor influencing cLac. The applied biological testing highlights the capability of ANN to capture complex nonlinear interactions among acid–base parameters, providing a valuable tool for clinical data interpretation and supporting precision monitoring in critical care. These findings confirm that the ANN models not only perform well statistically but also respect the physiological constraints inherent to arterial blood gas analysis. The biological testing phase demonstrates that the neural network captures meaningful biomedical patterns rather than relying solely on mathematical correlations, supporting its potential for clinical application and decision support.

## 5. Conclusions

Machine learning techniques are useful in creating the models that indicate the factors influencing the ventilation parameters. The ANN and LASSO regression indicated the most significant factors influencing pH, pCO_2_, pO_2,_ and cLac. The created ANN models were well-trained and avoided overfitting. The correlation coefficients exceeded the value of 0.92. In the case of LASSO regression, it excluded the insignificant values and indicated that the most significant variables for pH were pCO_2_, cCl^−^. In the case of pO_2_, it was FiO_2_ and pO_2_/FiO_2._ In the case of cLac, it was pCO_2_ and pH. Similar factors were pointed out in the sensitivity analysis conducted for ANN. For pCO_2,_ it was pH, cNa^+^, and cCl^−^. The ANN indicated Fshunt and pO_2_/FiO_2_ as the most significant. The difference may be the result of the characteristics of the model. ANN is based on the nonlinear model, contrary to LASSO regression, which is linear and eliminates the non-significant variables. Variables with higher sensitivity values in the ANN are more critical contributors to the analyzed parameters and should be prioritized when interpreting acid–base status or in clinical monitoring. Lower-ranked variables might represent indirect or modulatory effects on pH and may be less impactful independently but still relevant in a combined physiological context. LASSO regression indicated that pH and pCO_2_ models are dominated by their reciprocal acid–base predictors. In the case of pO_2_, it is strongly influenced by oxygen saturation and delivery metrics. cLac is predicted by a broad spectrum of interacting variables, illustrating its clinical complexity.

In recent years, more and more papers have been published indicating the potential application of machine learning techniques in medicine. Geng et al. described the potential use of ANNs in predicting the occurrence of hypoxemia during sedation, based on the patient’s BMI, snoring, and neck circumference [49]. Radhakrishnan et al. [4] developed the model using an MLP to predict ventilation parameters. According to the study of Vaghefi et al. [10], the promising tool for analysis are the multidimensional neural maps. They enhance prediction performance due to their capability to represent complex, high-dimensional data structures visually and cluster data effectively. Incorporating such networks could improve feature extraction and dimensionality reduction prior to regression modeling, or act as standalone predictive models.

The applied techniques in the study such as ANNs and LASSO regression offer complementary advantages for clinical data analysis. LASSO regression simplifies models by selecting only the most important variables. It enhances interpretability and reduces overfitting. ANNs provide powerful nonlinear modeling and sensitivity analysis that reveal priorities among clinical parameters and help to exclude the not significant ones. Together, they offer a robust framework combining high predictive performance with transparent information on variable importance.

For clinicians, it implies a better ability to quickly identify critical factors affecting ventilation parameters such as pH, pCO_2_, pO_2_, and cLac. High-sensitivity variables indicated by ANN should be prioritized clinically. LASSO’s variable selection helps exclude less relevant factors. This integrated approach supports faster, more reliable decision-making and increases understanding of complex physiology of acid–base balance and respiratory system, ultimately facilitating patient monitoring and therapy optimization. Machine learning thus has potential to transform clinical practice by efficiently analyzing large datasets and revealing key physiological insights.

The analyses conducted using machine learning techniques demonstrate the feasibility of using regression models to analyze the influence of individual factors on the values of arterial and venous blood gas parameters. Combining LASSO with ANN provides a robust framework that leverages the high predictive power of ANN while preserving model simplicity and offering insight into variable importance through regularization. They allow us to analyze the degree of influence of individual independent variables on dependent variables and to determine whether it is significant. Neural networks and LASSO regression allow the prediction of events based on the data provided. Machine learning techniques are useful in analysis of a large amount of information. They can help to estimate the impact of individual variables, which can improve clinical decision-making, and it allows for a significant shortening of time needed to study and understand pathophysiology.

## Figures and Tables

**Table 1:** The gasometric parameters for arterial blood.

Variable	Mean ± SD	Median	Minimum	Maximum
FiO_2_ [%]	44.28 ± 20.86	40.00	21.00	100.00
pH	7.42 ± 0.09	7.43	6.91	7.59
pCO_2_ [mmol/L]	41.16 ± 8.36	40.75	2.70	83.30
pO_2_ [mmHg]	115.76 ± 42.94	108.00	6.90	308.00
ctHb [g/dL]	11.49 ± 2.46	11.10	6.80	41.40
sO_2_ [%]	97.75 ± 4.08	99.00	68.00	100.0
cK^+^ [mmol/L]	3.73 ± 0.60	3.70	1.90	7.30
cNa^+^ [mmol/L]	141.15 ± 4.97	140.00	126.00	159.00
cCl^−^ [mmol/L]	105.72 ± 6.34	105.00	89.00	125.00
cLac [mmol/L]	1.49 ± 1.95	0.90	0.30	17.00
pO_2_/FiO_2_ [mmHg]	310.91 ± 163.33	320.00	32.80	1234.00
Fshunt [%]	11.63 ± 11.13	8.10	0.00	52.20

**Table 2:** The correlation analysis between pH, pCO_2_, pO_2_, cLac, and the independent variables for arterial blood.

Variable	FiO_2_	pH	pCO_2_	pO_2_	ctHb	sO_2_	cK^+^	cNa^+^	cCl^−^	cLac	pO_2_/FiO_2_	Fshunt
pH	−0.189	1.000	−0.489	−0.021	0.047	0.211	−0.354	−0.137	−0.310	−0.421	0.173	−0.215
pO_2_	−0.074	−0.021	−0.121	1.000	−0.132	0.567	0.016	−0.032	0.014	−0.001	0.591	−0.533
pCO_2_	0.233	−0.489	1.000	−0.121	0.009	−0.196	0.098	0.042	−0.237	−0.117	−0.352	0.284
cLac	0.182	−0.421	−0.117	−0.001	−0.021	−0.241	0.382	0.152	0.198	1.000	−0.118	0.198

**Table 3:** The results of the sensitivity analysis for the variables analyzed.

pH		pO_2_		pCO_2_		cLac	
Variable	Rank	Variable	Rank	Variable	Rank	Variable	Rank
cCl^−^	4.0085	pO_2_/FiO_2_	29.7513	Fshunt	8.8687	pH	3.0156
pCO_2_	3.9872	FiO_2_	22.6468	pO_2_/FiO_2_	4.5329	pCO_2_	2.4469
cNa^+^	2.1979	Fshunt	2.8196	cCl^−^	3.9112	Fshunt	2.0705
cLac	1.9464	sO_2_	1.1220	pH	3.5341	pO_2_/FiO_2_	1.8647
pO_2_/FiO_2_	1.3188	pH	1.0571	cNa^+^	2.2918	FiO_2_	1.8644
sO_2_	1.2463	pCO_2_	1.0312	FiO_2_	2.1653	cK^+^	1.5499
Fshunt	1.2354	cCl^−^	1.0248	cLac	1.6521	cCl^−^	1.4644
FiO_2_	1.1535	cNa^+^	1.0085	sO_2_	1.5504	sO_2_	1.4076
pO_2_	1.1392	cK^+^	1.0074	pO_2_	1.1285	pO_2_	1.2642
cK^+^	1.1102	cLac	1.0035	ctHb	1.1140	cNa^+^	1.1774
ctHb	1.0208	ctHb	1.0026	cK^+^	1.0577	ctHb	1.1193

**Table 4:** The coefficients for LASSO regression for arterial blood.

pH (λ = 0.0645)		pO_2_ (λ = 0.0504)		pCO_2_ (λ = 0.0270)		cLac (λ = 0.0642)		
Factor	Coefficient	Factor	Coefficient	Factor	Coefficient	Factor	Coefficient	
Intercept	0.0079	Intercept	0.1062	Intercept	−0.0288	Intercept	−0.0251	
pCO_2_	−1.4616	FiO_2_	0.4827	pH	−0.2298	FiO_2_	0.0384	
pO_2_	−0.0058	pH	−0.0640	cNa^+^	0.2124	pH	−0.3354	
cK^+^	−0.0693	pCO_2_	0.0171	cCl^−^	−0.2316	pCO_2_	−0.6246	
cNa^+^	0.3990	sO_2_	0.3066	cLac	−0.1132	sO_2_	−0.1030	
cCl^−^	−0.5592	cCl^−^	−0.0115	pO_2_/FiO_2_	−0.0821	cK^+^	0.1440	
cLac	−0.3799	pO_2_/FiO_2_	1.0699			cNa^+^	0.0396	

## Data Availability

The original contributions presented in this study are included in the article. Further inquiries can be directed to the corresponding author.

## Abbreviations

The following abbreviations are used in this manuscript:

ANN	Artificial Neural Network
ARDS	Acute respiratory distress syndrome
cCl^−^	Chloride concentration
cK^+^	Concentration of potassium
ctHb	Total concentration of hemoglobin
cLac	Concentration of lactate
cNa^+^	Sodium concentration
FiO_2_	Fraction of inspired oxygen
Fshunt	Fraction of the measured transpulmonary shunt
Hb	Hemoglobin
LASSO	Least Absolute Shrinkage and Selection Operator
MLP	Multilayer Perceptron
pCO_2_	Partial pressure of CO_2_
PEEP	Positive end-expiratory pressure
pO_2_	Partial pressure of O_2_
sO_2_	Saturation
